# Supplementary material for: Low-Platinum-Content Exchange-Coupled CoPt Nanoalloys with Enhanced Magnetic Properties
Source: Nanomaterials (Basel). 2024 Mar 7;14(6):482. doi: 10.3390/nano14060482 (PMC10974076; doi:10.3390/nano14060482)
Supplement: Supplementary file 1 [file nanomaterials-14-00482-s001.zip › nanomaterials-2887396-supplementary.pdf]

Supplementary Material

Low platinum content exchange-coupled CoPt nanoalloys with enhanced magnetic properties

Georgia Basina [1,3]*, Vasileios Alexandrakis [1], Ioannis Panagiotopoulos [2], Dimitrios Niarchos [1,5], Eamonn Devlin [1], Margarit Gjoka [1], George C. Hadjipanayis [3,4] and Vasileios Tzitzios [1,*]

1 Institute of Nanoscience and Nanotechnology, National Centre for Scientific Research, "Demokritos", 15310, Athens, Greece
2 Department of Materials Science and Engineering, University of Ioannina, GR, 45110, Ioannina, Greece
3 Department of Physics and Astronomy, University of Delaware, Newark, DE 19716, USA
4 Department of Chemical Engineering, Northeastern University, Boston, MA, USA
5 Amen New Technologies, Athens, Greece

* Correspondence: g.basina@inn.demokritos.gr; v.tzitzios@inn.demokritos.gr

Abstract: Bimetallic colloidal CoPt nanoalloys, with low platinum content, were successfully synthesized following a modified polyol approach. Powder X-ray diffraction, (XRD), fourier-transform infrared spectroscopy, (FT-IR), thermogravimetric analysis, (TGA), and transmission electron microscopy, (TEM), studies were performed to estimate the crystal structure, morphology, and surface functionalization of the colloids respectively, while the room temperature magnetic properties were measured using vibrating sample magnetometer (VSM). The particles exhibit excellent uniformity, with a narrow size distribution, and display strong room temperature hysteretic ferromagnetic behavior even in the as-made form. Upon annealing at elevated temperatures, progressive formation and co-existence of exchange coupled both chemically ordered and disordered phases, significantly enhance the room temperature coercivity.

Keywords: cobalt-platinum alloy; $L1_0$ phase; CoPt; Co_3Pt; exchange coupling; bimetallic nanoparticles; polyol method

CONTENT:

Supplementary material contains structural, magnetic and morphological characterization data based on XRD, VSM, TEM, Rietveld analysis as well as selected literature data on the room temperature coercivity values of CoPt-based nanomaterials, are given for comparison.

- **Figure S1.** Size distribution histograms of as-made (a) and annealed at 700 °C/7h (b) CoPt nanoparticles.
- **Figure S2.** Rietveld analysis of CoPt nanoalloys annealed at 700 °C/7h.
- **Figure S3**. M vs. 1/H curve of CoPt nanoalloys annealed at 700 °C/7h.
- **Figure S4.** Magnetic hysteresis loop of the annealed at 700 °C for 2h sample, and the 1st derivative of M vs H curve.
- **Figure S5.** Room temperature hysteresis loops of CoPt nanoparticles after annealing at 675 °C for 1.5h (a) and 4h (b).
- **Figure S6**. HAADF STEM image of the 7 h annealed CoPt nanoparticles (a) and elemental mapping of Co (red), Pt (green).
- **Figure S7.** Powder XRD pattern (a), and room temperature magnetic hysteresis loop (b), of equiatomic bimetallic CoPt nanoalloys, after annealing under reducing atmosphere at 700 °C for 4h.
- **Table S1.** Lattice parameter, space group and crystal structure of the CoPt nanoalloys annealed at 700 °C/7h.
- **Table S2.** Selected literature data on the room temperature coercivity values of various CoPt-based nanoalloys, for comparison.

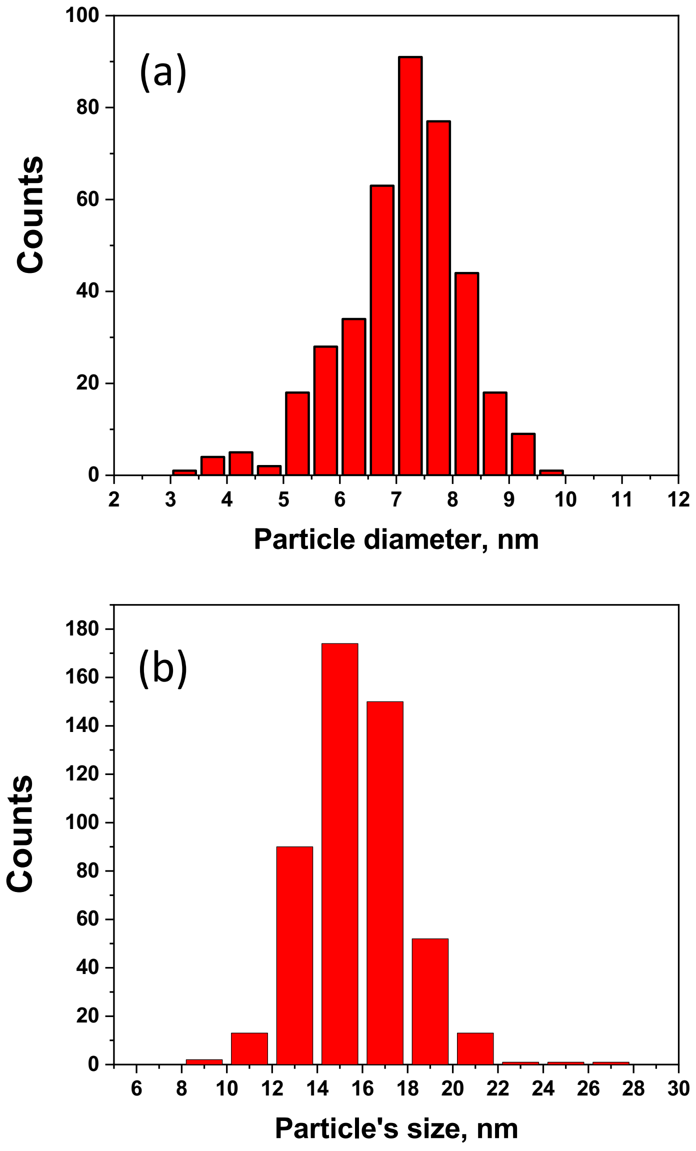

Figure S1. Size distribution histograms of as-made (a) and annealed at 700 °C / 7h (b) CoPt nanoparticles.

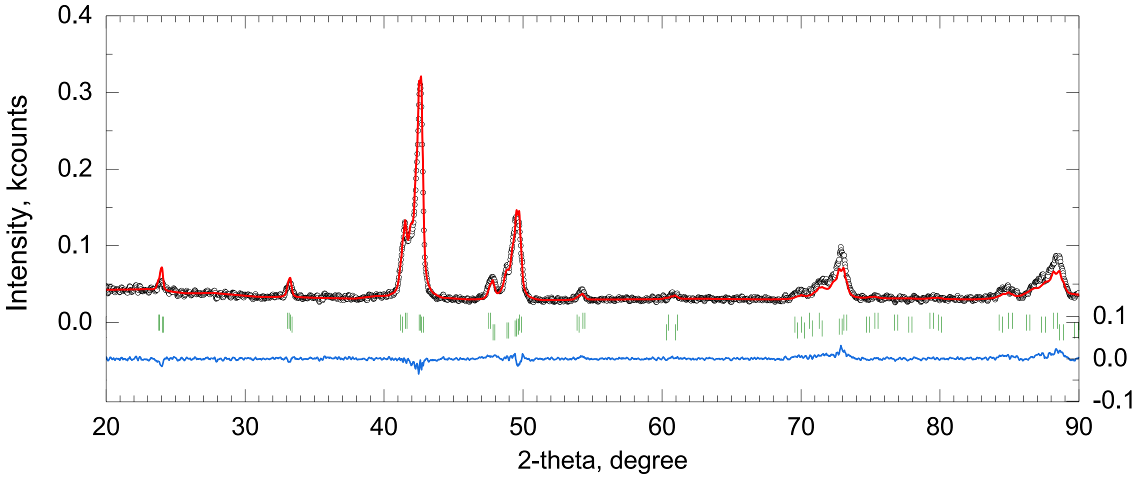

Figure S2. Rietveld analysis of CoPt nanoalloys annealed at 700 °C/7h

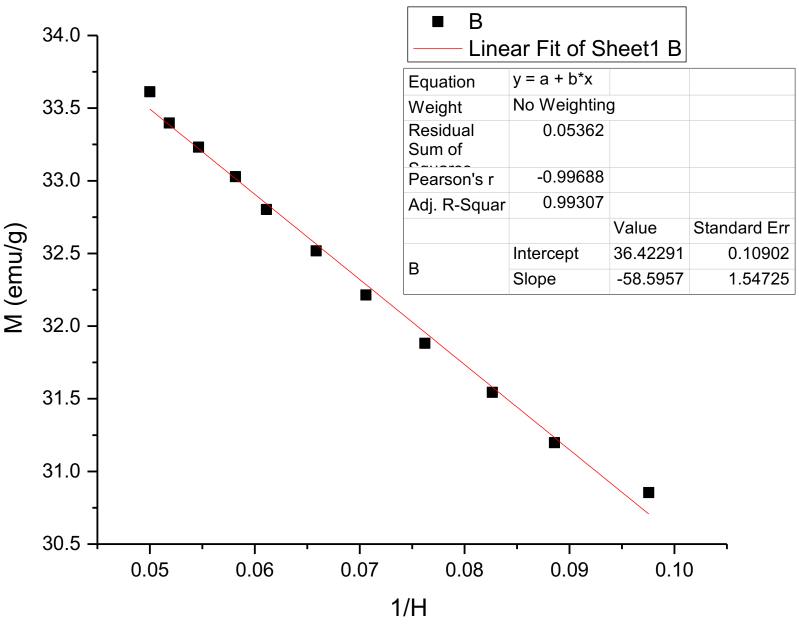

Figure S3. M vs. 1/H curve of CoPt nanoalloys annealed at 700 °C / 7h.

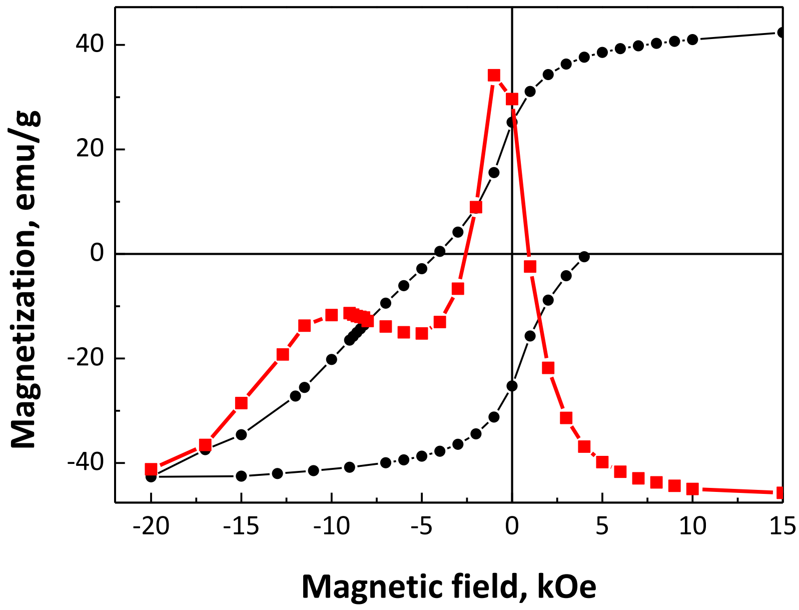

Figure S4. Magnetic hysteresis loop of the annealed at 700 °C for 2h sample, (*black line and symbols*), and the 1st derivative of M vs H curve (*red line and symbols*). The derivative curve is shifted for sake of visualization.

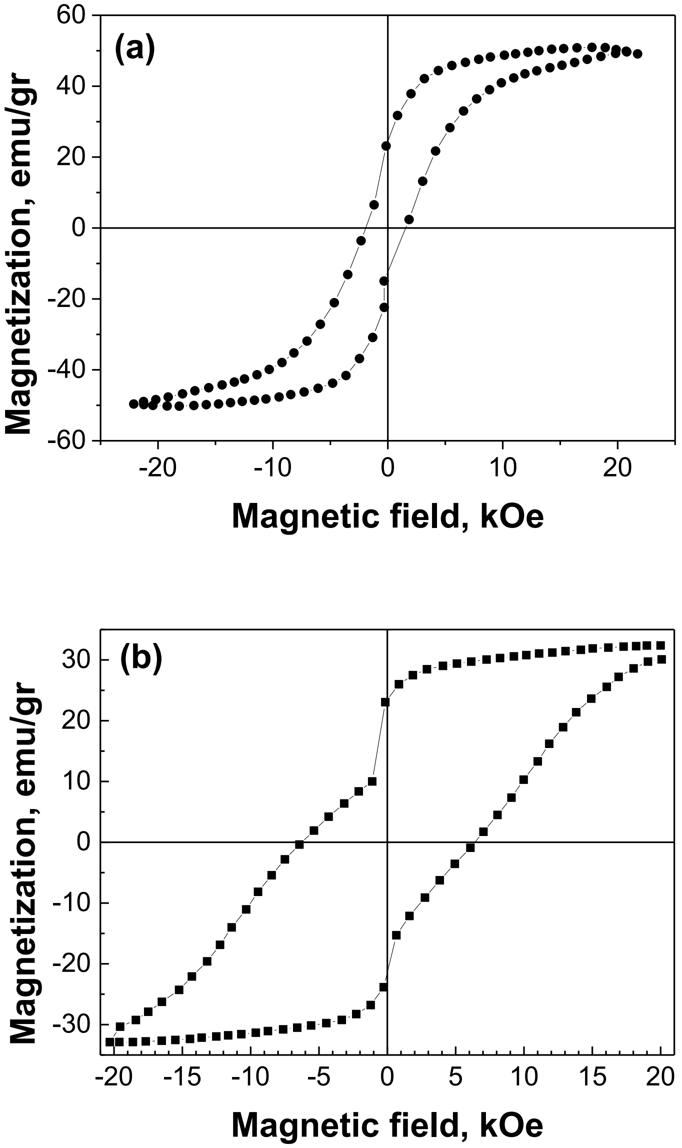

Figure S5. Room temperature hysteresis loops of CoPt nanoparticles after annealing at 675 °C for 1.5h (a) and 4h (b).

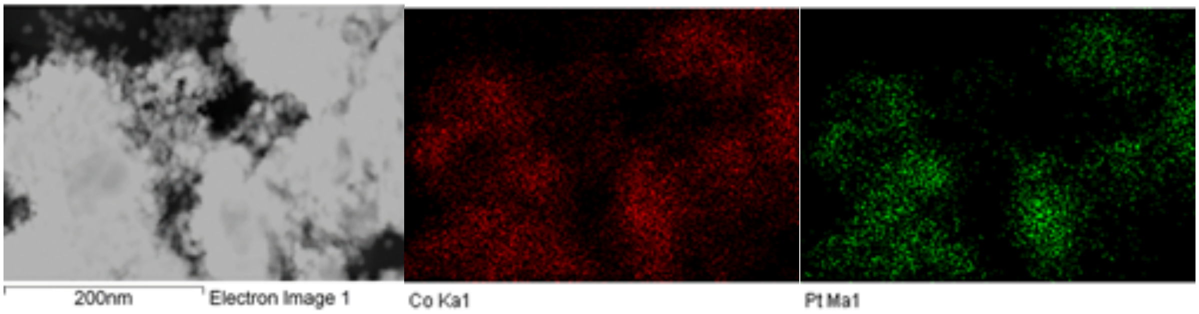

Figure S6. HAADF STEM image of the 7 h annealed CoPt nanoparticles (a) and elemental mapping of Co (red), Pt (green).

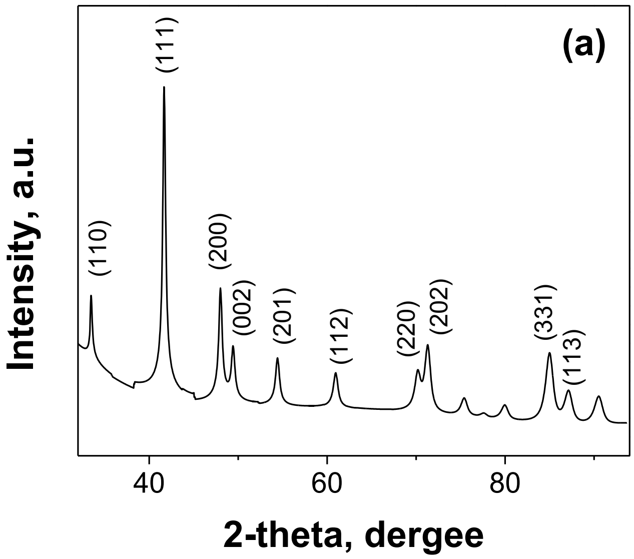

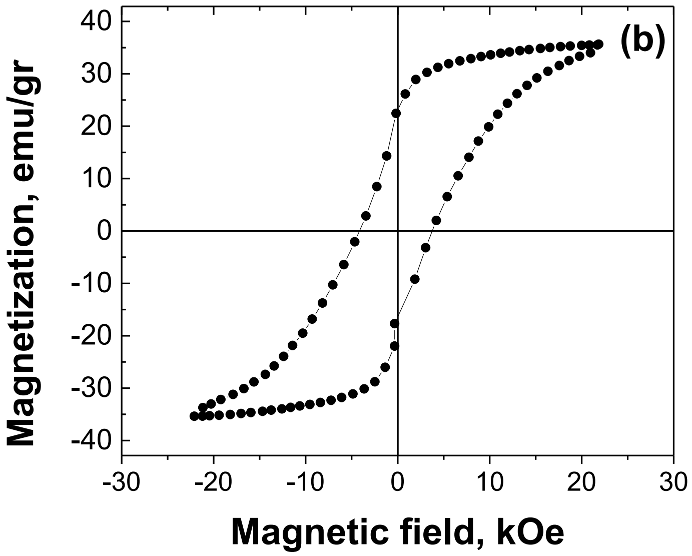

Figure S7. XRD pattern (a), and room temperature magnetic hysteresis loop (b), of equiatomic bimetallic CoPt nanoalloys, after annealing under reducing atmosphere at 700 °C for 4h.

Table S1

Lattice parameter, space group and crystal structure of the CoPt nanoalloys annealed at 700 °C/7h.

	CoPt P4/mmm tetragonal	CoPt P4/mmm tetragonal	Co_3Pt Fm-3m cubic	Co_3Pt Fm-3m cubic
a (Å)	3.79	3.83	3.65	3.72
b (Å)	2.68	2.71	3.65	3.72
c (Å)	3.68	3.73	3.65	3.72

Table S2

Selected literature data on the room temperature coercivity values of various CoPt-based nanoalloys, for comparison.

**Material**	**Annealing Conditions**	**Coercivity at RT, kOe**	***Particle Diameter, nm***	***Reference***
CoPt	650 °C and 700 °C, 1h	12 and 10.7	-	[1]
Core-shell CoPt	laser annealing (650 K)	0.1	6	[2]
CoPt	700 °C, 3h	0.63	-	[3]
CoPt	665 °C, 30 min	9 at 5K	-	
CoPt	800 °C	5.4	3.4	[4]
CoPt	550 °C	3.2	-	[5]
CoPt core-shell	700 °C, 12h	5.3	-	[6]
CoPt	650 °C	4	11	[7]
CoPt	700 °C	7.57	>>4	[8]
CoPt	700 °C	6	18	[9]
CoPt	800 °C	4.442	18	[10]
CoPt on Carbon	650 °C, 6h	7.1	8.9	[11]
CoPt, Bismuth addition	700 ^{o}C, 1h	14.5	29	[12]

REFERENCES

1. Sun, X.; Jia, Z.Y.; Huang, Y.H.; Harrell, J.W.; Nikles, D.E.; Sun, K.; Wang, L.M. Synthesis and magnetic properties of CoPt nanoparticles. *Journal of Applied Physics* **2004**, *95*, 6747-6749.
2. Bigot, J.-Y.; Kesserwan, H.; Halté, V.; Ersen, O.; Moldovan, M.S.; Kim, T.H.; Jang, J.-t.; Cheon, J. Magnetic Properties of Annealed Core–Shell CoPt Nanoparticles. *Nano Letters* **2012**, *12*, 1189-1197.
3. Chen, M.; Nikles, D.E. Synthesis of spherical FePd and CoPt nanoparticles. *Journal of Applied Physics* **2002**, *91*, 8477-8479.
4. Dong, Q.; Qu, W.; Liang, W.; Guo, K.; Xue, H.; Guo, Y.; Meng, Z.; Ho, C.-L.; Leung, C.-W.; Wong, W.-Y. Metallopolymer precursors to L10-CoPt nanoparticles: synthesis, characterization, nanopatterning and potential application. *Nanoscale* **2016**, *8*, 7068-7074.
5. Bian, B.; He, J.; Du, J.; Xia, W.; Zhang, J.; Liu, J.P.; Li, W.; Hu, C.; Yan, A. Growth mechanism and magnetic properties of monodisperse $L1_0$-Co(Fe)Pt@C core–shell nanoparticles by one-step solid-phase synthesis. *Nanoscale* **2015**, *7*, 975-980.
6. Park, J.-I.; Kim, M.G.; Jun, Y.-w.; Lee, J.S.; Lee, W.-r.; Cheon, J. Characterization of Superparamagnetic "Core−Shell" Nanoparticles and Monitoring Their Anisotropic Phase Transition to Ferromagnetic "Solid Solution" Nanoalloys. *Journal of the American Chemical Society* **2004**, *126*, 9072-9078.
7. Wellons, M.S.; Gai, Z.; Shen, J.; Bentley, J.; Wittig, J.E.; Lukehart, C.M. Synthesis of $L1_0$ ferromagnetic CoPt nanopowders using a single-source molecular precursor and water-soluble support. *Journal of Materials Chemistry C* **2013**, *1*, 5976-5980.

8. Chinnasamy, C.N.; Jeyadevan, B.; Shinoda, K.; Tohji, K. Polyol-process-derived CoPt nanoparticles: Structural and magnetic properties. *Journal of Applied Physics* **2003**, *93*, 7583-7585.

9. Tzitzios, V.; Niarchos, D.; Gjoka, M.; Boukos, N.; Petridis, D. Synthesis and Characterization of 3D CoPt Nanostructures. *Journal of the American Chemical Society* **2005**, *127*, 13756-13757.

10. Zhang, Y.J.; Yang, Y.T.; Liu, Y.; Wang, Y.X.; Yang, L.L.; Wei, M.B.; Fan, H.G.; Zhai, H.J.; Liu, X.Y.; Liu, Y.Q.; et al. A novel approach to the synthesis of CoPt magnetic nanoparticles. *Journal of Physics D: Applied Physics* **2011**, *44*, 295003.

11. Li, J.; Sharma, S.; Liu, X.; Pan, Y.-T.; Spendelow, J.S.; Chi, M.; Jia, Y.; Zhang, P.; Cullen, D.A.; Xi, Z.; et al. Hard-Magnet L10-CoPt Nanoparticles Advance Fuel Cell Catalysis. *Joule* **2019**, *3*, 124-135.

12. Abel, F.M.; Basina, G.; Tzitzios, V.; Alhassan, S.M.; Sellmyer, D.J.; Hadjipanayis, G.C. Ferromagnetic $L1_0$-Structured CoPt Nanoparticles for Permanent Magnets and Low Pt-Based Catalysts. *ACS Applied Nano Materials* **2021**, *4*, 9231-9240.
